# Application of Moringa leaves as soil amendment to tiger-nut for suppressing weeds in the Nigerian Savanna

**DOI:** 10.1186/s12870-023-04170-6

**Published:** 2023-04-10

**Authors:** Olasupo James Fadeyi, Thomas Oladeji Fabunmi, Adeniyi Adebowale Soretire, Victor Idowu Olugbenga Olowe, Adeyemi Olusegun Raphael

**Affiliations:** 1 Institute of Food Security, Environmental Resources and Agricultural Research (IFSERAR), Federal University of Agriculture, (FUNAAB), Abeokuta, Ogun State Nigeria; 2 Department of Plant Physiology and Crop Production, FUNAAB, Abeokuta, Ogun State Nigeria; 3 Department of Soil Science and Land Management, FUNAAB, Abeokuta, Ogun State Nigeria

**Keywords:** Allelopathy, Bio-herbicide, *Cyperus esculentus*, Moringa leaves, Tuber size, Weed – management

## Abstract

**Background:**

The allelopathic effect of Moringa (*Moringa oleifera *Lam.) leaves applied as organic manure in tiger nut (*Cyperus esculentus* L.) production on associated weeds was investigated in the guinea savanna of South West Nigeria, during the 2014 (September - November) and 2015 (June - August) wet seasons.

**Methods:**

Five Moringa leaves rates (0, 2.5, 5.0, 7.5 and 10 t/ha) and three tuber sizes (0.28 g, 0.49 g and 0.88 g dry weight) were laid out in the main plot and sub-plot, respectively in a split-plot arrangement fitted into randomized complete block design and replicated three times.

**Results:**

Parameters measured, which include, weed cover score (WCS), weed density (WD) and weed dry matter production (WDMP) were significantly (p<0.05) influenced in both years by Moringa leaf. In 2015, WCS, WD and WDMP significantly (p<0.05) reduced by 25–73%, 35–78% and 26–70% on Moringa leaves-treated plots respectively. There were significant (p<0.05) interactions between quantity of Moringa leaves incorporated and tuber size. The bigger the tuber and the higher the quantity of Moringa leaves incorporated the lower the WCS, WD and WDMP.

**Conclusions:**

Consequently, application of 10 t.ha^− 1^ Moringa leaves and planting of large or medium-sized tubers were recommended for optimum weed suppression in tiger nut production in South West Nigeria.

## Introduction

Tiger nut (*Cyperus esculentus*) is a crop that has received considerable attention of food scientists due to its nutritional and health benefits. Its milk can serve as substitute for traditional cow milk. The edible, stable and superior oil obtained from the tuber of tiger nut compares favourably with olive oil, and it has also been used in treatment of flatulence, indigestion, diarrhoea, dysentery and excess thirst [1]. Moringa (*Moringa oleifera*) is an important plant of Moringaceae family with a range of medical uses and nutritional value; it contains a profile of important minerals, protein, vitamin, Beta carotene, amino acids and various phenolics [2]. The leaves, roots, seed, bark, fruit, flowers and immature pods act as cardiac and circulatory stimulants; possesses antitumor and other important health values [2]. In addition to the numerous importance of Moringa, it also has tremendous allelopathic potential either to suppress or stimulate the neighbouring crop [3]. These allelopathic effects have been documented by various researchers [4–8]. Allelopathy refers to any process involving secondary metabolites produced by plants, micro-organisms, fungi etc. that positively or negatively influence the growth and development of agricultural and biological systems and these secondary metabolites or allelochemicals are transferred through the environment from one organism to the other being released into the environment (atmosphere or rhizosphere) in ample quantities by means of volatilization, leaching, decomposition of residues, root exudation etc. and if persistent long enough could either stimulate or inhibit the growth and physiological processes of the neighbouring or successional plant [9]. It has also been noted that at low allelochemical concentrations, plants commonly exhibit stimulatory allelopathic effect on its neighbors, whereas high concentrations can suppress the growth and germination of nearby plants [10]. Allelopathic potentiality of Moringa under field conditions can be utilized in different ways such as, surface mulch, incorporation into the soil, aqueous extracts, rotation, smothering or mix cropping/intercropping [11[12]. reported that in general, leaves are the most potent source of allelochemicals, although, the toxic metabolites are also distributed in all other plant parts in various concentrations. They reported that allelochemicals released from plants are useful for weed management options in several agriculture settings to reduce dependency on commercial herbicide which has been found to be hazardous. Herbicidal effects of *Moringa oleifera* on the germination and seedling survival of *Euphorbia heterophylla* L. [5]; *Amaranthus spinosus* [6]; inhibitory effects of *Moringa oleifera* on the growth of broad and grassy weeds associated with *Narcissus tazetta* L. [7]; herbicidal actions of *Moringa oleifera* on sunflower associated grassy weed *Econocloa colonum* [8] have therefore been reported. Given this trend, there are no reports of studies conducted on both the text crop and in this ecology. This study therefore, evaluated the inhibitory effect of Moringa leaves applied as organic manure in tiger nut production on associated weeds in the Guinea Savanna agro-ecological zone of South West Nigeria.

## Materials and methods

The trial was conducted during 2014 (September - November) and 2015 (June – August) in the Federal University of Agriculture Abeokuta. The location was at the Institute of Food Security, Environmental Resources and Agricultural Research (IFSERAR) of the University, Opeji (7^0^13’51.17’’ – 7^0^13’53.16’’N, 3^0^23’49.12’’ – 3^0^23’51.86’’E) in the Southern Guinea Savannah Agro-ecological zone. The experiment was laid out in a split-plot design with three replications. The main plots and sub plots were manure rates and the tuber sizes, respectively. The Moringa leaves rates were 0, 2.5, 5.0, 7.5 and 10 t/ha; while the three tuber sizes: small, medium and large had dry weights of 0.28 g, 0.49 and 0.88 g, respectively per tuber. There were 45 experimental units; each plot measured 2 m x 1 m; there was 50 cm within a replication and 1 m between blocks. Yellow variety of tiger nut tubers used for the trials was purchased from the local market, graded and then soaked in water for 24 h to enhance sprouting. Tillage operations included ploughing, harrowing and bed construction. After land preparation, fresh moringa leaves were incorporated at the main plot treatment rates once prior to planting of tiger nut in 2014 cropping season according to the experimental design and treatments. In 2015, Tigernut was planted on the same beds used for 2014 trial after tilling, with residue of previous year’s application. In year 2014, the trial was carried out during the late season (September - November). Planting was done on the 3rd September. The inter and intra row spacings were 50 cm and 25 cm, respectively to give a plant population of 80,000 plants/ha. In year 2015, the trial was conducted during the early season (June - August). After the usual soaking of the tubers for 24 h, the tubers were pre-sprouted in saw dust for 8 days prior to field establishment. Planting was done on 6th June. Manual weeding was carried out at 3 and 6 weeks after planting (WAP). Weed cover was observed at 6 WAP and the score from 0 to 10 was given to each observation from no weed cover (0) to complete weed cover (10). Weed density, which is the number of weeds per unit area (1 m^2^) was counted at 6 WAP using 1 × 1 m quadrat. For weed dry matter production, weed samples were collected at 6 WAP from 1 m^2^ quadrat in each plot, before weeding exercise, the weeds were dried to a constant weight and then weighed with a sensitive scale. Data collected were subjected to analysis of variance (ANOVA) using SPSS package and significant means were separated using Duncan’s Multiple Range Test (DMRT) at 5% level of significance.

## Results and discussion

### Soil physical and chemical properties of the experimental site

The sandy-loam soil with pH slightly acidic is preferable for tiger nut production. N, P and K, were low but were optimally enhanced by the Moringa leaves application (Table 1).

**Table 1:** The Pre-treatment and Post-treatment Physico-chemical properties of the soil of the experimental site

Parameters	Pre-treatment	Post-treatment
Physical properties		
Sand (%)	83.2	85.2
Silt (%)	10.6	7.6
Clay (%)	6.2	7.2
Textural class	Sandy-loam	Sandy-loam
Chemical properties		
pH	5.76	5.92
Organic carbon (%)	0.7	3.36
Organic matter (%)	1.21	5.79
N (%)	0.07	0.34
Available P (mg/kg)	0.9	7.54
Ca (cmol/kg)	11.95	6.88
Mg (cmol/kg)	0.34	0.26
Na (cmol/kg)	0.28	0.3
K (cmol/kg)	0.17	0.34
CEC (cmol/kg)	12.86	7.89

### Nutrient composition of Moringa leaves

The percentage nitrogen was 4.1 while the carbon to nitrogen ratio (C: N) ratio was 2.6. Other macro nutrients, in order of magnitude were calcium (12.93), magnesium (1.3) and potassium (0.32) with phosphorus (0.0087) as the least (Table 2).

**Table 2:** Nutrient composition of Moringa foliage

Nutrient	Values
Organic carbon (%)	10.8
N (%)	4.10
C:N	2.6
P (%)	0.0087
K (%)	0.32
Ca (%)	12.93
Mg (%)	1.3
Na (%)	2.0

### Growth conditions

More rainfall was recorded in 2014 although relatively lower in this ecology. Relative humidity, temperature as well as sunshine hours were optimum in both years (Table 3).

**Table 3:** Meteorological data during the experiment in 2014 and 2015 at Opeji

Month	Total Rainfall (mm)		Relative Humidity (%)		Temperature ^0^ C				Sunshine Hours	
					Maximum		Minimum			
	2014	2015	2014	2015	2014	2015	2014	2015	2014	2015
June	-	12.8	-	78.6	-	29.2	-	25.8	-	2.8
July	-	0.0	-	70.8	-	28.6	-	25.9	-	4.1
August	-	9.2	-	89.8	-	28.3	-	25.3	-	4.2
September	56.8	-	96.2	-	29.6	-	27.1	-	3.4	-
October	16.7	-	91.4	-	27.8	-	25.6	-	5.7	-
November	4.5	-	95.9	-	30.7	-	26.3	-	6.1	-

Source: Oyo State College of Agricultural Technology, Igboora. Oyo State

### Effects of moringa leaf rates and seed tuber size on weed cover score, weed density and weed dry matter production in tiger nut in 2014 and 2015

Moringa incorporation had significant (p < 0.05) effect on WCS, WD and WDMP in both years (Table 4). In 2014, the effect was not in a definite pattern except on WCS. On all Moringa - treated plots WCS was lower (p < 0.05) than untreated plots (Table 4). This indefinite pattern can be attributed to slow release of the secondary metabolites or allelochemicals embedded in the Moringa leaves being an organic substance. However, in 2015, Moringa leaves incorporated reduced WCS, WD and WDMP by 25–73%, 35–78% and 26–70% respectively on Moringa leaves-treated plots (Table 4). These reductions could be attributed to the suppressive effect of Moringa leaves applied owing to the allelopathic effect of the Moringa leaves corroborated by earlier studies [5–8]. The higher the quantity of leaves applied the more the reduction (Table 4). This could be due to the fact that at higher concentrations, allelochemicals from allelopathic plants can suppress the growth and germination of nearby plants [10]. In the earlier reports, [6] concluded that, the higher the concentration of Moringa crude water leaf extract applied the more the reduction in germination and growth of *Amaranthus spinosus*; while [5] similarly concluded from his investigation that the seedling survival of *Euphorbia heterophylla* L. got reduced as the concentration of the fresh leaf extract of Moringa increased. Seed tuber size gave varied results only on WCS in 2014 (Table 4); large and medium tuber sizes had similar WCS on their plots which were significantly lower (p < 0.05) than WCS on small tuber size plots (Table 4). Quantity of Moringa leaves incorporated and tuber size also had significant interactions on the WCS, WD and WDMP in 2015 trial only (Table 4).

**Table 4:** Effects of Moringa leaf rates and Seed tuber size on Weed Cover Score, Weed Density and Weed Dry Matter Production at 6 WAP of Tigernut at Opeji, 2014 and 2015

Treatments	2014			2015		
	W CS	W D (g/m^2^)	W D M P (g)	W CS	W D (g/m^2^)	W D M P (g)
Manure rate (tha-1) (M)						
0	4.8 c	29.6 b	30.7 b	7.6 a	37 a	36.6 a
2.5	6.7 ab	33.6 b	30.0 b	5.7 b	24 b	26.9 b
5.0	7.0 a	45.3 a	41.3 a	4.3 c	21 c	20.0 c
7.5	6.0 ab	45.9 a	31.7 b	4.0 c	17 d	14.3 d
10.0	6.7 ab	34.2 b	33.5 b	2.0 d	8 e	11.0 e
S.E±	1.2	1.6	0.8	0.3	1.3	1.4
F test	*	*	*	*	*	*
Seed tuber size (T)						
SMALL	5.6 b	38.1	32.9	4.9	22	22.3
MEDIUM	6.6 a	36.2	33.4	4.5	21	21.7
LARGE	6.5 a	38.8	34.0	4.7	21	21.2
SE±	0.2	1.6	0.8	0.3	1.3	1.4
F test	*	ns	ns	ns	ns	ns
Interaction						
MXT	ns	ns	ns	**	**	**

*In a column, means followed by similar letter are not significantly different at 5% level of probability using Duncan’s Multiple Range Test (DMRT). WCS Weed Cover Score. WD = Weed Density. WDMP = Weed Dry Matter Production. ns = not significant.* = Significant at 5% level of probability.

### Interaction of quantity of Moringa leaves incorporated and seed tuber size on weed cover score weed density and weed dry matter production

**Fig. 1:**
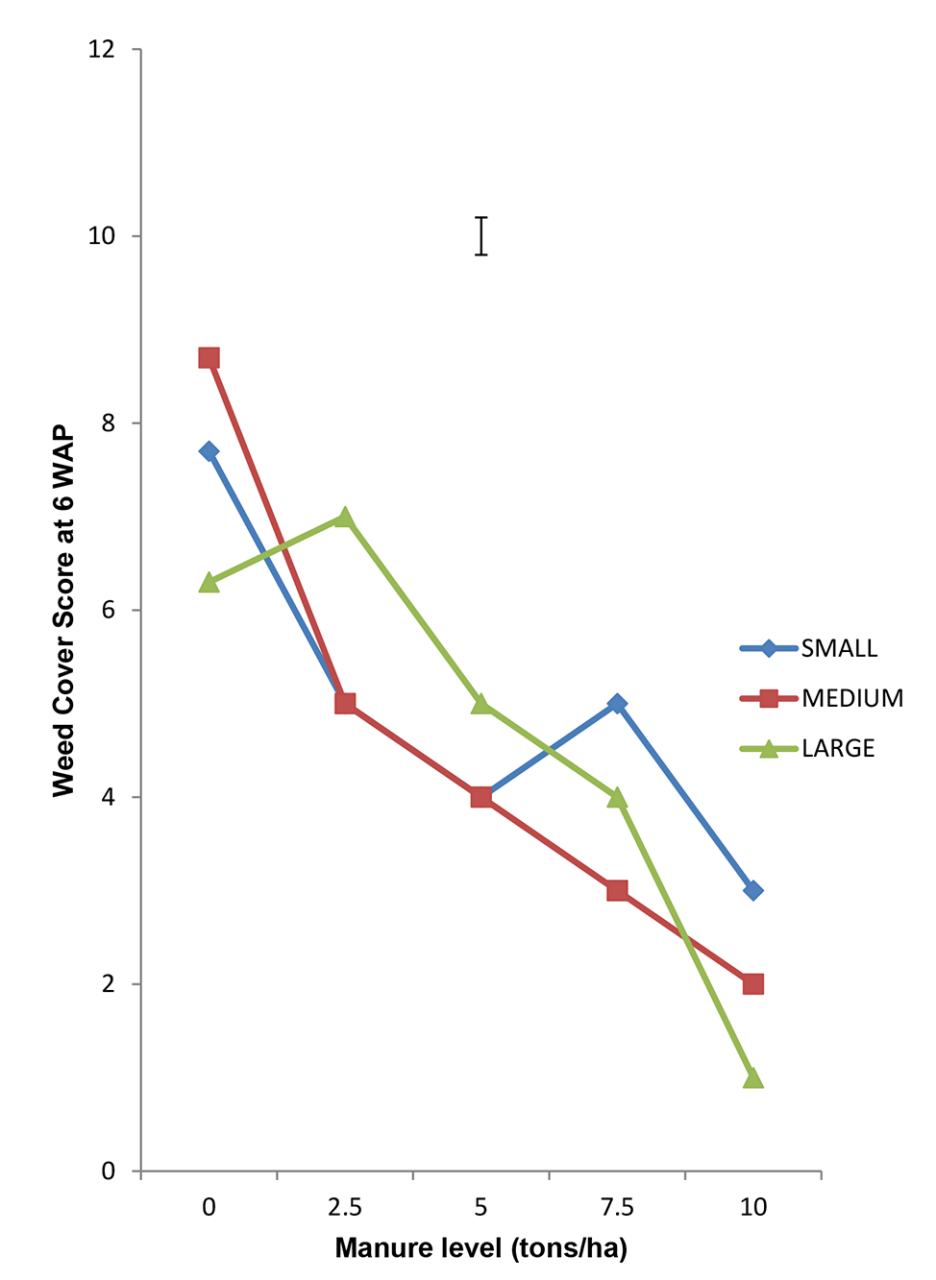
Interaction of Moringa Foliage and Seed tuber size on Weed Cover Score at 6 WAP of Tigernut at Opeji, 2015 Cropping Season

**Fig. 2:**
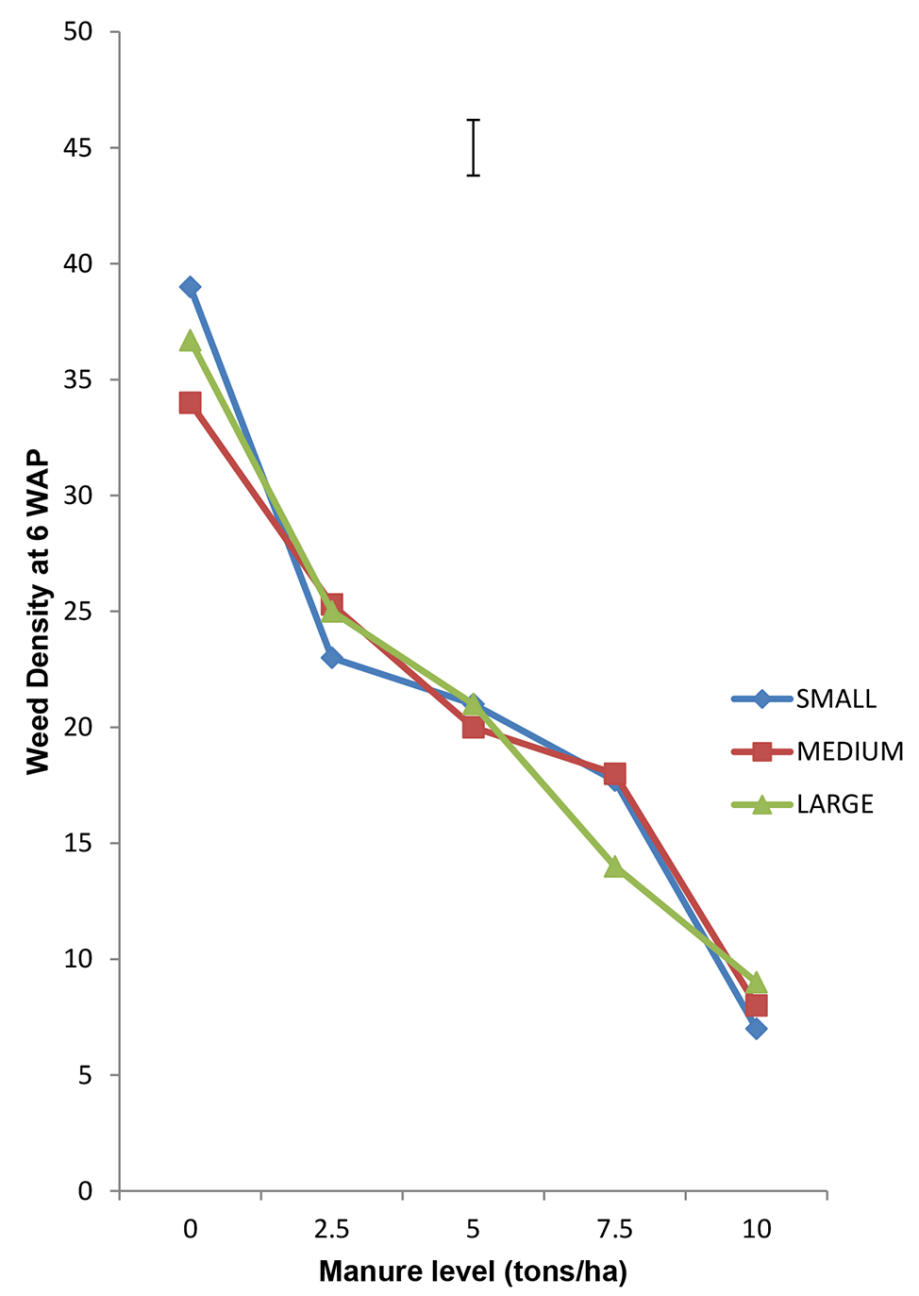
Interaction of Moringa Foliage and Seed tuber size on Weed Density at 6 WAP of Tigernut at Opeji, 2015 Cropping Season

**Fig. 3:**
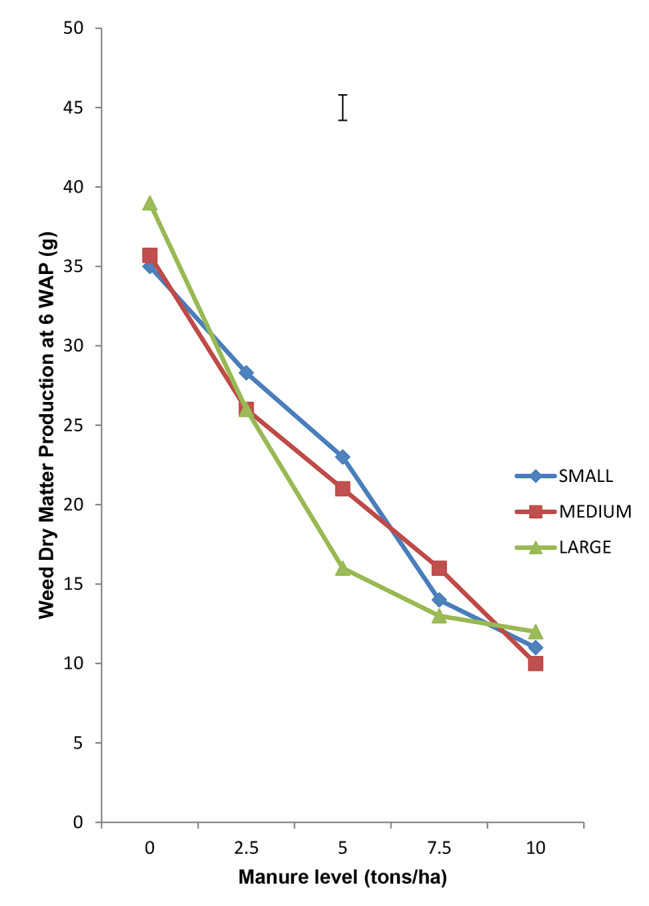
Interaction of Moringa Foliage and Seed tuber size on Weed Dry matter Production at 6 WAP of Tigernut at Opeji, 2015 Cropping Season

Interaction of Moringa leaf rate and tuber size on WCS, WD and WDMP revealed that the bigger the tuber and the higher the quantity of Moringa leaves incorporated, the lower the WCS, WD and WDMP (Figs. 1, 2 and 3 respectively). This synergy suggests that bigger tubers possessed more food reserve which enhanced better growth to produce more vigorous seedlings to compete actively and smother weeds relative to the less vigorous seedlings. The resultant effect therefore, is that they complement better to the suppressive actions of the released allelochemicals from the incorporated Moringa leaves which increased with higher rates.

## Conclusion

Growth and survival of weeds were controlled by the application of Moringa leaves. Bigger tubers complemented the inhibitory effects of higher rates of Moringa leaves and thus, were incorporated better for weed suppression relative to smaller tubers. Incorporation of 10 t. ha^− 1^ foliage produced lower WCS, WD and WDMP than other rates. Moringa leaf is a viable option as bio-herbicide in tiger nut production. It can be concluded that for maximum weed suppression, 10 t.ha^− 1^of Moringa leaves should be incorporated into the soil and large or medium-sized tubers should be planted.

## Data Availability

The datasets used and/or analysed during the current study available from the corresponding author on reasonable request.
